# Interaction of the mechanosensitive microswimmer *Paramecium* with obstacles

**DOI:** 10.1098/rsos.221645

**Published:** 2023-05-24

**Authors:** Nicolas Escoubet, Romain Brette, Lea-Laetitia Pontani, Alexis Michel Prevost

**Affiliations:** ^1^ Sorbonne Université, CNRS, Institut de Biologie Paris-Seine, Laboratoire Jean Perrin, 4 place Jussieu, Paris 75005, France; ^2^ Sorbonne Université, INSERM, CNRS, Institut de la Vision, 17 rue Moreau, Paris 75012, France

**Keywords:** microswimmer, mechanosensitivity, *Paramecium*, avoiding reaction, micropillars

## Abstract

In this work, we report investigations of the swimming behaviour of *Paramecium tetraurelia*, a unicellular microorganism, in micro-engineered pools that are decorated with thousands of cylindrical pillars. Two types of contact interactions are measured, either passive scattering of *Paramecium* along the obstacle or avoiding reactions (ARs), characterized by an initial backward swimming upon contact, followed by a reorientation before resuming forward motion. We find that ARs are only mechanically triggered approximately 10% of the time. In addition, we observe that only a third of all ARs triggered by contact are instantaneous while two-thirds are delayed by approximately 150 ms. These measurements are consistent with a simple electrophysiological model of mechanotransduction composed of a strong transient current followed by a persistent one upon prolonged contact. This is in apparent contrast with previous electrophysiological measurements where immobilized cells were stimulated with thin probes, which showed instantaneous behavioural responses and no persistent current. Our findings highlight the importance of ecologically relevant approaches to unravel the motility of mechanosensitive microorganisms in complex environments.

## 1. Introduction

In their natural habitat, microorganisms evolve in complex environments that are characterized by the presence of obstacles with different shapes, sizes and mechanical properties. Therefore, foraging motile microorganisms have to swim efficiently in response to various stimuli such as chemical or light gradients. In this context, ciliated microorganisms have the ability to sense the properties of their environment through mechanosensitive processes [1–4]. *Paramecium* in particular is a large unicellular eukaryote organism (100−300 μm long) whose entire surface is covered with thousands of cilia that beat synchronously [5]. When it hits an obstacle, the mechanosensitive channels at its front open and trigger an avoiding reaction (AR): *Paramecium* swims backwards for a short time, then reorients and swims forward in a new direction [6]. On the contrary, when *Paramecium* is poked on its posterior end, it displays an escape reaction during which it accelerates forward, a response that is elicited by a different type of mechanosensitive channel [3]. The behaviour of *Paramecium* in complex environments is therefore dependent on its mechanosensitivity.

At the cellular level, it is known that a mechanical stimulation opens mechanosensitive channels located in the plasma membrane, which are specifically permeable to calcium [7–9]. The resulting inward calcium current depolarizes the membrane, which then opens voltage-gated calcium channels in the cilia, triggering an action potential and the reversal of the cilia [10,11]. Mechanotransduction has been previously studied using electrophysiology experiments [2,3]. However, in these experiments, the mechanical stimulation was systematically applied with an external probe and not elicited upon swimming. Conversely, in behavioural studies, the swimming behaviour of *Paramecium* in the presence of obstacles has been reported qualitatively but not systematically quantified [1], except for the special cases of microfluidic channels [12,13], sliding along surfaces [14,15] or during direct interactions between paramecia [16].

In this paper, we study the swimming behaviour of *Paramecium* in controlled crowded environments and focus on its local interactions with obstacles. In recent years, similar approaches have been developed for a different microorganism, the microalga *Chlamydomonas reinhardtii* (CR), which swims through the synchronized beating of two front flagella. On long timescales, the presence of dense arrays has been shown to decrease the effective diffusivity of CR [17] or deflect their trajectories [18]. On short timescales, the local interactions of CR with either flat or curved surfaces have been studied and characterized through both contact and hydrodynamic modelling [19–21]. However, none of these studies has explicitly taken into account the mechanosensitive properties of the microswimmer in the modelling of their interactions with surfaces.

In our study, we distinguish between the hydrodynamic interactions and the contact regime that can lead to a mechanosensitive response. For interactions that do not lead to an AR, we recover a behaviour that has been reported previously for CR [20]: when *Paramecium* contacts a pillar, it is scattered with a fixed angle, while when it interacts without contact, its trajectory is linearly deflected. When an AR is elicited upon contact, we show that the mechanosensitive behaviour can be separated into two distinct responses: an AR that is triggered immediately upon contact with the obstacle, and an AR which is delayed. We then show that both responses can be accounted for by a simple model where a mechanotransduction current is integrated during contact until an excitability threshold is reached. The model accounts for all our observations, but in contrast to previous studies on immobilized cells stimulated by moving rods [3,22], it predicts that with an ecological stimulus, the mechanotransduction current is small and has a persistent component.

## 2. Results

### 2.1. Interactions with a pillar

**Figure 1.**
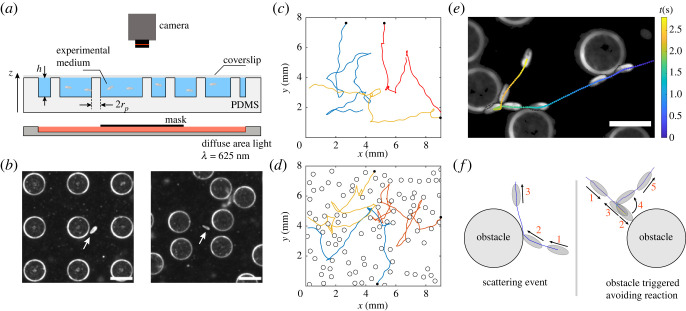
Tracking *Paramecium* in micro-engineered environments. (*a*) Sketch of the experimental set-up (side view). Paramecia are deposited in a PDMS elastomer pool filled with the experimental medium (see Material and methods) and decorated with cylindrical pillars (height *h* = 340 μm, diameter 2*r*_*p*_ ≃ 300 μm). The pool is covered with a glass coverslip to prevent evaporation. Paramecia are illuminated in dark field with a red LED panel (*λ* = 625 nm) and imaged from the top with a fast and sensitive camera. (*b*) Typical dark-field images of paramecia (white arrows) swimming in a square lattice (left panel, surface packing fraction Φ=0.2) and in a random lattice (right panel, Φ=0.083). The scale bar is 300 μm long. (*c*,*d*) Two-dimensional trajectories of three paramecia in a pool, (*c*) without obstacles and (*d*) with obstacles (Φ=0.28, random). For each trajectory, the black disc marks its starting point. (*e*) Composite image showing a close-up view of the trajectory of a paramecium swimming in a random lattice of obstacles (Φ=0.14). The colour of the trajectory codes for the elapsed time and a few successive positions of the same paramecium at different times are overlayed. The scale bar is 300 μm long. (*f*) Sketch of the two types of interactions with a pillar: (left) scattering event (SE), (right) obstacle-triggered avoiding reaction event (OTAR). On both sketches, the black arrows indicate the direction of motion and the numbers the successive steps of the events.

Typical experiments consist of tracking *Paramecium tetraurelia* swimming in elastomer-based pools whose bottom is either smooth or decorated with cylindrical obstacles of radius *r*_*p*_ ≃ 150 μm, spatially distributed on random and square lattices with surface fractions Φ ranging from 0.011 to 0.28, where Φ is defined as the ratio between the sum of all pillar top surfaces and the total area of the pool (see table 1, Material and methods and electronic supplementary material). As shown in figure 1*a*, the height of the pillars matches the depth of the pool *h* = 340 μm and paramecia are thus constrained to the volume between the pillars and cannot swim above them. In addition, since the depth of the pool is approximately three times the length of *Paramecium*, the cells can swim helicoidally but their trajectories are mostly constrained in two dimensions. Some reorientation events associated with ARs can actually occur perpendicularly to the observation plane (in approximately 21% of the cases for *ϕ* = 0, see electronic supplementary material for details). Figure 1*b* shows two examples of such micro-engineered pools (a square and a random lattice) imaged with a dark field illumination, so that both the edges of the pillars and paramecia appear as bright objects on a darker background (see white arrows on both images to locate the paramecia).

**Table 1.** Environments used in this work. The first column provides the lattice type. The other ones give the pillar surface fraction Φ and number *M* of experiments performed (brackets). In addition, 10 experiments were done without pillars (Φ=0) to observe the free swimming of the cells.

square	0.083 (8)	0.14 (13)	0.20 (9)	0.28 (9)
random	0.011 (13)	0.083 (6)	0.14 (10)	0.28 (7)

Without obstacles, the normal motion of *Paramecium* alternates between helicoidal swimming runs and spontaneous reorientations, as shown in figure 1*c* with the example of three typical trajectories. These events have been coined ‘avoiding reactions’ (AR) as they usually occur in response to different types of stimuli, such as obstacles, a local change of temperature or the presence of attractants and/or repellents in the environment [1,6]. They may also occur spontaneously due to membrane potential fluctuations [23]. During an AR, *Paramecium* comes to a stop, swims backward for a short period of time and then performs a three-dimensional rotation of its body around its posterior end, before resuming its forward swimming motion in a new direction (see electronic supplementary material for the detailed dynamics of the AR, section III, figure S3 and movie S1). It results at long times in a motion (figure 1*c*) that is thus reminiscent of the classical *run and tumble* swimming dynamics of *Escherichia coli* bacteria [24].

As first reported by Jennings more than a century ago [1], an AR can also be triggered mechanically when *Paramecium* comes into direct contact with an obstacle, as depicted on the trajectories of figure 1*d*, obtained for paramecia swimming in a random network. In this case, the AR probably results directly from the opening of mechanosensitive channels located in the plasma membrane of *Paramecium*. However, contacts with an obstacle clearly do not only induce such triggered AR, but also lead to reorientation events during which *Paramecium* is ‘passively’ scattered by the obstacle. Figure 1*e* shows a typical trajectory for which *Paramecium* first slides against two pillars at early and intermediate times (in blue), exhibiting passive-like scattering events (see electronic supplementary material, section V, figure S4 and movie S2), and then performs an AR upon hitting a third pillar (see the green to yellow coloured points of the trajectory in figure 1*e*). For the rest of the manuscript, we will refer to the first two events of figure 1*e* as scattering events (or SE) and to the last one as an obstacle-triggered avoiding reaction (or OTAR). Both types of events are sketched in figure 1*f*. Note that in what follows, results obtained with both types of lattice structures were pooled, as we did not observe any differences in the statistics of the reorientation events.

### 2.2. Passive interactions

**Figure 2.**
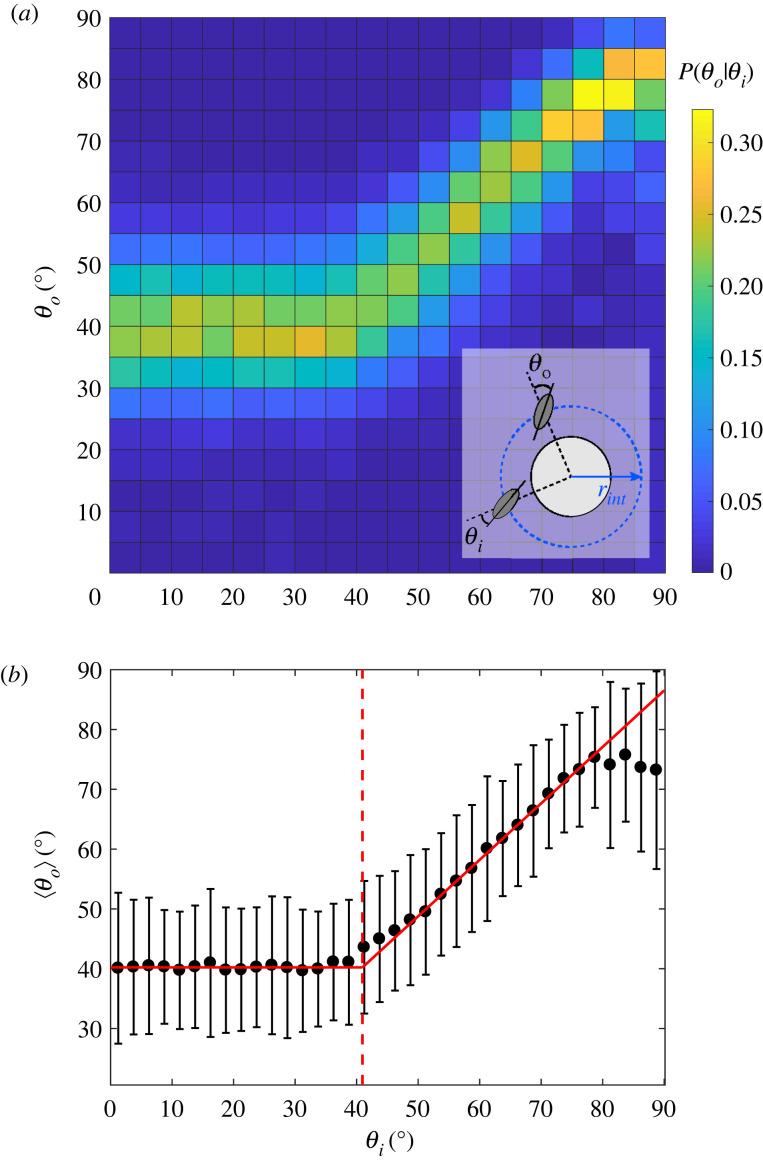
Characterizing passive interactions. (*a*) Conditional probability *P*(*θ*_*o*_|*θ*_*i*_) for *θ*_*i*_ and *θ*_*o*_ varying between 0° and 90°. Each square bin has a side length of 5°. This plot was obtained from *N* = 28 031 events compiled from *M* = 75 independent experiments ranging from *ϕ* = 0.01 to *ϕ* = 0.28 in a square or random lattice. Inset: sketch to define the geometrical parameters. The blue dashed circle corresponds to the interaction corona while the solid black circle corresponds to the pillar. A paramecium intercepts the interaction corona with an angle *θ*_*i*_ and leaves it with the angle *θ*_*o*_. (*b*) Mean output angle 〈*θ*_*o*_〉 versus *θ*_*i*_, where *θ*_*i*_ is taken as the centre value of each bin, of width 2.5°. Solid red lines are linear fits of the contact and hydrodynamic regimes, that intercept at $\;{\tilde{\;\theta }}_{i}=41^{\circ }$, marked by the vertical red dashed line. In the hydrodynamic regime, the linear fit yields, for *θ*_*i*_ ∈ [50°, 80°], a slope *m* = 0.94 ± 0.031 and intercept *q* = 1.6 ± 2.0° with *R*^2^ = 0.99.

How does *Paramecium* passively interact with an obstacle? To quantify this, we have followed the same approach as Kantsler *et al.* [19] and Contino *et al.* [20] by studying how the cells interact with a pillar depending on their incident angle, in the absence of any AR. For this purpose, we define an interaction corona as the circular region of radius *r*_int_ = 256 μm and centred on the pillar, thus yielding a ring of width approximately one cell length around the pillar. We denote *θ*_*i*_ (*resp.* *θ*_*o*_) the angle between the local radial direction and the orientation of the cell when it enters (*resp.* leaves) the interaction corona (figure 2*a*, inset).

We have measured *θ*_*i*_ and *θ*_*o*_ over *N* = 28 031 passive interaction events, i.e. events during which the cell has travelled through the interaction corona without any AR, recorded from *M* = 75 independent experiments (figure 2*a*). Below a threshold incidence angle $\;{\tilde{\theta }}_{i}=41^{\circ }\pm 3.7^{\circ }$, cells always leave the pillars with the same mean angle $\left\langle \;{\tilde{\theta }}_{o}\right\rangle \approx 40^{\circ }\pm 0.22^{\circ }$. In this case, there is no memory of the in-going angle *θ*_*i*_. Above $\;{\tilde{\theta }}_{i}$, the cell travels through the interaction corona without actually contacting the pillar. In that regime, its trajectory is simply deflected and the mean angle 〈*θ*_*o*_〉 depends linearly on *θ*_*i*_ with a slope *m* = 0.94 ± 0.031 (figure 2*b*). The measured value of $\;{\tilde{\theta }}_{i}$ matches a simple geometric prediction, based on a tangent contact of the cell with the pillar, $\;{\tilde{\theta }}_{i}=\arcsin\left((r_{\;p}+b)/r_{\mathrm{int}}\right)=42^{\circ }\pm 1.3^{\circ }$ with the semi-width of the cell *b* = 29.5 ± 4.4 μm.

The measurements for the contact regime are coherent with our observations that upon contact, the cell first reorients and aligns along the pillar, then slides against it before leaving tangentially to the pillar, independently of the in-going angle. Finally, the conditional probability map *P*(*θ*_*o*_|*θ*_*i*_) found for *Paramecium* is very similar to the one measured by Contino *et al.* [20] with the biflagellate microalga CR, suggesting a universal behaviour at contact among microswimmers, independently of their swimming mode. In the following, we will focus exclusively on the interactions during which the cell collides mechanically with the obstacle and we will thus not consider the hydrodynamic regime, for which no actual mechanical contact occurs between the cells and the pillars.

### 2.3. Obstacle-triggered avoiding reactions

Although it is well known that mechanical stimulation of the front part of *Paramecium* can trigger an AR [1,25], it is unclear how ARs are elicited upon contact on larger obstacles such as our pillars, and at the swimming speed of *Paramecium* itself. We first ensured that ARs in the vicinity of obstacles were indeed triggered by the mechanical contact with the obstacle, i.e. that they are OTARs and not spontaneous ARs happening to be close to it by chance. To do so, we looked at the radial dependence of the AR frequency *f*_AR_(*r*) defined as

**Figure 3.**
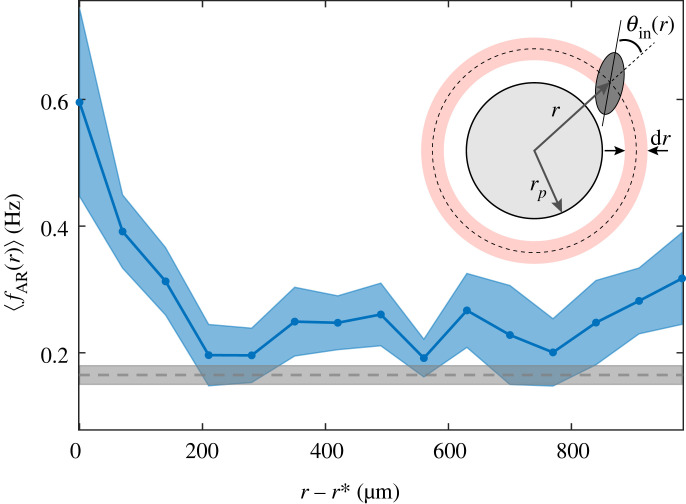
Detecting ARs in the vicinity of a pillar. Average AR frequency 〈*f*_AR_(*r*)〉 at a distance *r* − r* from the surface of the pillar. The blue curve is obtained by averaging *f*_AR_(*r*) from *M* = 13 independent experiments and the blue-shaded area corresponds to the standard error of the mean (s.e.m.). The experiments were performed in a randomly distributed pillar array with Φ=0.01. The horizontal grey dashed line corresponds to the mean avoiding reaction frequency $\left\langle f_{\mathrm{AR}}^{\Phi =0}\right\rangle =0.165$ Hz in the absence of obstacles and computed over *M* = 10 independent experiments, and the grey shaded area gives the s.e.m. Inset: sketch to illustrate the geometrical parameters. For these measurements, a bin size d*r* = 70 μm was chosen, that matches the average semi-length of a cell.

2.1 $$f_{\mathrm{AR}}(r)=\frac{n_{\mathrm{AR}}(r\pm dr/2)}{T_{\mathrm{obs}}(r\pm dr/2)},$$

where *n*_AR_ is the total number of AR in the annulus of width d*r* at position *r*, and *T*_obs_ is the total observation time of cells in this annulus (see inset in figure 3).

Figure 3 shows 〈*f*_AR_(*r*)〉 plotted as a function of the distance from the surface of the pillar *r* − r*, with r* = *r*_*p*_ + *b* ∼ 172 μm the inaccessible volume radius, and where the brackets denote an averaging over *M* = 13 independent experiments. In the immediate vicinity of a pillar, one clearly sees that the spatial AR frequency increases by a factor of approximately 4 compared with its value far from the pillar, peaking at r*. Such an increase is a clear signature of a mechanosensitive response. In addition, far from the pillar, one recovers the mean value of the spontaneous AR frequency measured in pillar-free environments, $\left\langle f_{\mathrm{AR}}^{\phi =0}\right\rangle ≃0.165\;\mathrm{Hz}$.

**Figure 4.**
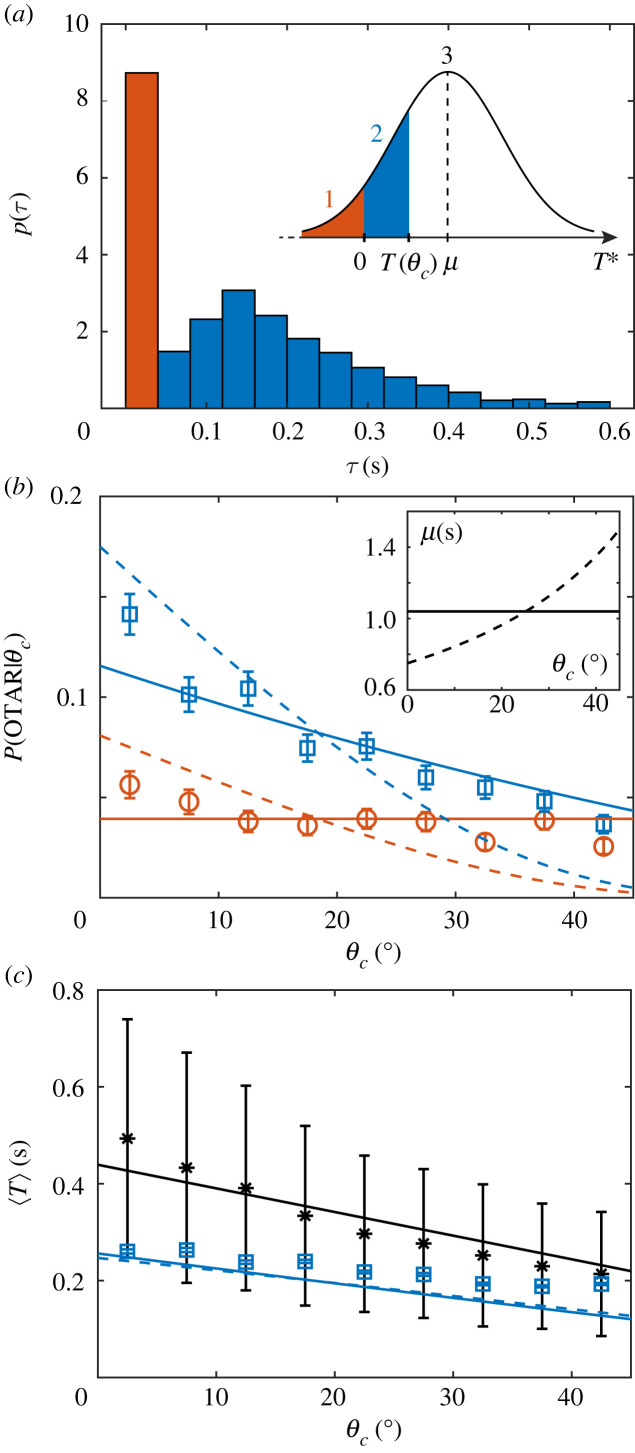
Identification and modelling of two types of OTARs. (*a*) Probability density function of the measured triggering time *τ* (bin width =0.04 s). Red (*resp.* blue) bars correspond to instantaneous (*resp.* delayed) OTARs. Inset: sketch of the model distribution of T* with mean μ, and cases 1, 2 and 3 defined in §2.4. (*b*) Probability of doing an instantaneous (red circles) or a delayed (blue squares) OTAR given the collision angle *θ*_*c*_, versus *θ*_*c*_ (bin width Δ*θ*_*c*_ = 5°). Error bar calculations are given in the Material and methods section. The solid (*resp.* dashed) line corresponds to the model with μ constant (*resp.* *θ*_*c*_ dependent, equation (2.6)): in red, *P*_1_ (equation 2.3); in blue, *P*_2_ (equation 2.4). Inset: μ versus *θ*_*c*_ for the uniform and non-uniform models (*resp.* solid and dashed lines). (*c*) Average contact duration 〈*T*(*θ*_*c*_)〉 for SEs (black stars) and delayed OTARs (blue squares). The solid black line is a linear fit of the SE data using equation (2.2), yielding *T*_max_ = 0.44 s (with *R*^2^ = 0.87). The solid (*resp.* dashed) blue line corresponds to the prediction of the contact duration *T*_2_ (equation 2.7) in the case of delayed OTARs with μ constant (*resp.* *θ*_*c*_ dependent).

Direct visual inspection of paramecia swimming against pillars reveals that not all OTARs are actually triggered instantaneously upon contact (see electronic supplementary material, movies S3 and S4). To quantify this effect, we have measured the duration between the collision time and the start of the backward swimming (BS) that marks the beginning of an OTAR (see electronic supplementary material and Material and methods for the determination of both times). This duration, noted *τ*, is called the triggering time. Figure 4*a* shows the distribution of *τ* obtained with a total of *N* = 1790 OTARs. This distribution has two peaks, a first narrow one at *τ* ≈ 0 s and a second broader one at *τ* ≈ 0.15 s. Thus, *Paramecium* displays two types of reactions: an immediate one, referred to as an *instantaneous OTAR* (*τ* ∈ [0, 0.04] s), and a delayed one, called *delayed OTAR* (*τ* ∈ ]0.04,0.6] s).

Furthermore, we find that the probability of an instantaneous OTAR does not depend on the contact angle *θ*_*c*_ (defined as the incident angle *θ*_in_ at contact, see inset of figure 3 and Material and methods), while the probability of a delayed OTAR decreases with increasing *θ*_*c*_ (figure 4*b*). We present below an electrophysiology-based model that accounts for these two distinct responses.

### 2.4. A simple model

How can *Paramecium* display two different types of behaviour upon contact, with a different dependence on the incidence angle? Electrophysiological studies on immobilized cells show that a mechanical stimulation of the front membrane triggers an inward calcium current with short latency and duration, in the millisecond range [3,22]. This transient current then depolarizes the membrane and quickly triggers an action potential, initiating the AR. Given this evidence, the observation of OTARs delayed by several hundreds of milliseconds is surprising. We propose here a simple model to explain both types of reactions. First, given that most contacts do not trigger an AR, we postulate that the current triggered upon contact is smaller than in typical mechanical stimulations of immobilized cells. To trigger an action potential, the charge *Q* transmitted at contact must exceed a threshold Q* ≈ CV*, where *C* ≈ 300 pF is the membrane capacitance [11] and V* ≈ 3 mV is the threshold potential to trigger an action potential relative to the initial potential, which quantifies cell excitability [26]. Thus, Q* ≈ 1 pC. Depending on the cell excitability at the instant of contact, the transmitted charge may or may not trigger an action potential.

Second, to account for the delayed reactions, we postulate that, in addition to the instantaneously transmitted charge, there is a small transduction current *I*_0_ that persists as long as the cell is in contact with the pillar. This could be due to an incomplete inactivation of the mechanosensitive channels, as observed in patch clamp recordings of *Piezo* channels [27], or to the progressive recruitment of mechanosensitive channels during sliding, as different parts of the membrane come into contact with the pillar. Therefore, during contact, a transmitted charge *Q* + *I*_0_*T* accumulates during a time *T* until it reaches the threshold Q* or the cell leaves the pillar. Thus, an AR is triggered if the cell remains in contact for a minimum duration T* = (Q* − *Q*)/*I*_0_. We assume that T* is a random variable normally distributed with probability density *p*, mean μ and standard deviation *σ*. This would occur for example if the membrane potential (which is noisy [23]) was normally distributed. Thus, we can see that three cases can occur: (1) for T* ≤ 0, a contact instantaneously triggers an AR: this is an instantaneous OTAR; (2) the cell stays in contact for a time T*, then does an AR: this is a delayed OTAR; (3) the cell leaves the obstacle before time T*: this is a scattering event SE.

To quantify the probability of each case, we first measured the duration *T*(*θ*_*c*_) of a SE as a function of the contact angle *θ*_*c*_ (figure 4*c*, black stars). If we assume, as our observations suggest, that the cell slides on the pillar until it leaves it tangentially, then this duration should depend approximately linearly on *θ*_*c*_ as follows:

2.2 $$T(\theta_{c})=T_{\max}\left(1-\left(\frac{\theta_{c}}{90}\right)\right).$$

Fitting this expression to our data (figure 4*c*, black solid line) yields *T*_max_ = 0.44 ± 0.035 s (95% confidence bounds, *R*^2^ = 0.87). Note that the same trend for *T*(*θ*_*c*_) was measured for *Pawn* cells, a mutant of *Paramecium tetraurelia*, which cannot perform ARs due to a lack of voltage-gated calcium channels in the cilia [28] (see electronic supplementary material, section VIII, figure S7). This confirms the passive nature of SEs.

We can now express the probability of each of the three cases (figure 4*a*, inset).

Case 1: An instantaneous OTAR occurs with probability
  2.3 $$P_{1}=\int_{-\infty }^{0}p(T^{*})\;dT^{*}$$
Case 2: A delayed OTAR occurs with probability
  2.4 $$P_{2}(\theta_{c})=\int_{0}^{T(\theta_{c})}p(T^{*})\;dT^{*}$$
Case 3: A SE occurs with probability
  2.5 $$P_{\mathrm{SE}}(\theta_{c})=\int_{T(\theta_{c})}^{+\infty }p(T^{*})\;dT^{*}.$$

The model has two free parameters, μ and *σ*, which we constrain with two experimental data points, *P*_1_(*θ*_*c*_ = 22.5°) and *P*_2_(*θ*_*c*_ = 22.5°). We find μ ≈ 1.04 s and *σ* ≈ 0.59 s, which means that it takes on average approximately 1 s for a contact to trigger an AR.

The model predicts that the probability of an instantaneous OTAR does not depend on *θ*_*c*_, in agreement with our observations (figure 4*b*, red solid line), because the transduction current is assumed to be independent of the contact position. Less intuitively, the model also predicts that the probability of a delayed OTAR decreases with increasing *θ*_*c*_, because contacts with smaller *θ*_*c*_ are longer. This angular dependence is in quantitative agreement with our observations (figure 4*b*, blue solid line).

Electrophysiological studies show that the transduction current vanishes in the middle of the cell (*θ*_*c*_ = 90°) (and reverts on the posterior side) [29]. What would happen if it decreased gradually over the anterior part with increasing *θ*_*c*_? We calculate the probabilities of the three cases with an angular dependence of the mean of T*, as follows:

2.6 $$\mu (\theta_{c})=\frac{c}{90-\theta_{c}},$$

where the constant *c* is chosen such that the angular average 〈μ(*θ*_*c*_)〉 equals the mean of T* in the uniform model (figure 4*b*, inset). Here, the triggering time T* is minimum at *θ*_*c*_ = 0° and infinite at *θ*_*c*_ = 90°. Note that for μ(*θ*_*c*_), we chose the simplest functional form in qualitative agreement with reported electrophysiological measurements, that meets the two boundary conditions: (i) μ is minimum at the front tip of the cell, where the mechanosensitive response is the highest, and (ii) μ is infinite at the lateral point of the cell, where there is no mechanosensitive response. Testing more precise functional forms would require controlled mechanical stimulations coupled with electrophysiology measurements, beyond the scope of the present work. Using equation (2.6), we evidence that the probabilities of both instantaneous and delayed OTARs vary substantially with the contact angle *θ*_*c*_ (figure 4*b*, red and blue dashed lines), unlike in the observations.

Finally, the model allows predicting the mean contact duration of delayed OTARs as a function of *θ*_*c*_, which is

2.7 $$T_{2}(\theta_{c})=E[T^{*}|0<T^{*}<T(\theta_{c})]=\frac{\int_{0}^{T(\theta_{c})}T^{*}p(T^{*})\;dT^{*}}{\int_{0}^{T(\theta_{c})}p(T^{*})\;dT^{*}}.$$

The prediction is in quantitative agreement with the observations (figure 4*c*, blue solid and dashed lines). These values are in the range of the measured secondary peak of the distribution of the triggering times *τ* (figure 4*a*). Even though the mean charging time is approximately 1 s, the triggering time is in fact limited by the maximum sliding duration against the pillars (approx. 0.3 s, figure 4*c*, blue squares).

## 3. Discussion

Motile microorganisms naturally evolve in complex environments that they have to sense and react to [30]. It is therefore critical to understand how their foraging efficiency depends on their local interactions with obstacles found in their natural habitat. In order to answer these questions, a large array of literature has been dedicated to the study of motility in engineered complex environments in the past years [17,20,21,31].

In this context, *Paramecium* is interesting, as it can be studied both through behavioural and electrophysiology approaches thanks to its large size. As a matter of fact, its swimming behaviour as well as its mechanosensitive response, have been extensively described in past literature, reviewed in [6]. The main message from these studies is that *Paramecium* performs an AR upon collision with an obstacle, through a mechanosensitive response, and this response is modulated by the location of the stimulation on the surface of the swimmer. However, our work reveals a different picture for the behaviour of this microorganism in our experimental conditions.

First of all, collision with an obstacle does not necessarily elicit an AR. In fact, it is passively scattered on the surface of the pillars 90% of the time, which means that its trajectory is deflected without exhibiting any backwards swimming. In this case, the scattering event imposes the angle *θ*_*o*_ at which the swimmer escapes the immediate neighbourhood of the pillar. Conversely, when *Paramecium* interacts hydrodynamicaly with the pillar, this angle *θ*_*o*_ depends linearly on the incident angle *θ*_*i*_. In the absence of an AR, our findings directly echo previous observations on a different microorganism [20], the microalgae *Chlamydomonas reinhardtii*.

The fact that only 10% of the contact events lead to an AR is surprising. Actually, a similar small rate of ARs (of less than 5%) has been reported by Ishikawa & Hota [16], although in a different context that involves collisions between two freely swimming paramecia. Apart from this particular case, such an observation has not been quantified in previous behavioural experiments of freely swimming paramecia, which were done more than a century ago [1]. Electrophysiological studies on immobilized cells on the other hand, did not suggest such a low rate of mechanical reactions [3,8,9,22]. This may be due simply to the fact that mechanical stimulation with probes was adjusted precisely so as to induce a measurable mechanosensitive response (see in particular fig. 3 in [3]). To our knowledge, all electrophysiological studies on the mechanotransduction of *Paramecium* used micrometre-sized glass probes, and it could well be that the applied forces were much stronger than in our experiments, especially as our obstacles are much wider than typical probes.

From a behavioural perspective, it is intriguing that ARs are relatively rare when *Paramecium* hits large obstacles such as our pillars. One possible explanation is that it might not be critical for the organism to react to a large obstacle when it can be passed by sliding. This would explain the occurrence of delayed reactions: an AR is triggered when the organism is blocked by the obstacle for a certain amount of time. Another possibility could be that mechanosensitivity serves not simply navigation in crowded environments, but also and perhaps primarily the avoidance of sharp objects that may harm the membrane, as is suggested by evolutionary accounts [32].

Another surprising finding is that many OTARs are delayed by approximately 150 ms, when electrophysiological studies report nearly instantaneous responses. According to voltage clamp measurements, mechanotransduction currents activate with a very short latency and a rise time smaller than 20 ms, in *Paramecium* and other ciliates [8]. In addition, the current appears to be transient. There are two possibilities to make our findings consistent with these previous studies. One is that the current inactivates only partially. Indeed, this is what has been observed in *Piezo* channels with a constant applied pressure [27]. Previous studies in *Paramecium* quantified the peak current but not its stationary value. Another possibility is that, as the organism slides along the obstacle, additional mechanosensitive channels are recruited.

Finally, another noticeable discrepancy with previous work is the distribution of mechanosensitivity along the body of *Paramecium*. While our experimental data is in agreement with a homogeneous distribution of mechanosensitivity along the front part of *Paramecium*, previous electrophysiological studies showed graded responses along the body axis [22]. Stimulating the anterior part triggers an inward current, carried mostly by calcium, while stimulating the posterior part triggers an outward current, carried by potassium. In the middle, the transduction current vanishes. When potassium channels are blocked pharmacologically, mechanical stimulation triggers a uniform depolarizing response across the body [3]. Thus, the spatial gradient of mechanosensitivity appears to be due to the cancellation of a spatially uniform depolarizing mechanoreceptor current by a spatially graded hyperpolarizing current. It is not entirely clear from previous measurements whether this cancellation is linear between the anterior and posterior end, and this is complicated by experimental difficulties. Indeed, immobilized cells were mechanically stimulated with a glass probe of fixed orientation, and with a fixed movement, while position along the membrane was varied. Thus, the orientation and direction relative to the membrane surface varied, and the amplitude of the membrane deflection was not fixed either. By contrast, in our experiments, the obstacles are very controlled in their shape and mechanical properties. Therefore, the contact only depends on the swimming behaviour of *Paramecium* itself. Moreover, it is unclear how the presence of electrophysiology micropipettes inside the organism and its immobilization might affect its mechanical properties. For instance, the presence of a pipette might impose a pre-stress on the cell surface. In fact, in artificial mechanoreceptors, wetting of the membrane on a pipette is sufficient to trigger the opening of mechanosensitive channels [33].

In the future, observing electrophysiological responses of swimming organisms to obstacles should therefore be key to unify findings obtained from electrophysiology and behavioural experiments.

## 4. Material and methods

### 4.1. Cell culture and preparation

Experiments were performed with the wild-type stock 51 of *Paramecium tetraurelia*, obtained from Eric Meyer, Institut de Biologie, Ecole Normale Supérieure, Paris, France. The cells were cultured in a fully opaque incubator set at 27°C in a buffered infusion of wheat grass powder (Wheat Grass Powder, Pines) complemented with 0.8 μg ml^−1^ *β*-sitosterol and inoculated with non-pathogenic *Klebsiella pneumoniae* as a food source. After 48 h of growth and at least 20 min before an experiment, approximately 0.4 ml of cell suspension were pipetted from the surface of the culture medium into 4 ml of an inorganic medium (1 mM CaCl_2_, 4 mM KCl and 1 mM Tris–HCl, pH 7.2). As a control, experiments with the *Pawn* mutant strain, which cannot perform ARs due to a lack of voltage-gated calcium channels in the cilia [28], were also performed. The same culture and preparation protocols were carried out.

### 4.2. Fabrication of the pools

Pools were manufactured using a combination of micro-milling and elastomer moulding techniques fully described in the electronic supplementary material, section I and figure S1. They are made of a polydimethylsiloxane elastomer (PDMS; Sylgard 184, Dow Corning; cross-linking ratio 10 : 1, Young’s modulus *E* ≃ 2.7 ± 0.8 MPa) and consist of a square wall of height *h* = 340 μm and edge length 30 mm that delimits an accessible volume. Its surface is either bare or decorated with cylindrical pillars of radius *r*_*p*_ = 142.5 μm, distributed according to a square lattice or randomly. For the square lattice, the surface fraction $\Phi =\pi r_{p}^{2}/s^{2}$ is varied by changing the mesh size *s*. For the random lattices, pillars are distributed randomly with a minimal spacing of approximately 60 μm to avoid trapping of the cells. Prior to an experiment, the elastomer pool was exposed to an oxygen plasma for approximately 1 min to render the PDMS surface hydrophilic. After injecting the cells, the pool was closed with a glass coverslip in contact with the top of the wall and the pillars.

### 4.3. Experimental set-up and imaging

Cells were imaged from the top with a variable zoom lens (MVL12X12Z, Thorlabs) set on 1.5× combined with a 1.33× extension tube (Thorlabs, MVL133A) yielding a pixel size of 3.81 ± 0.01 μm. Images were captured with a high-resolution and sensitive CMOS camera (Blackfly S BFS-U3-51S5M-C, Flir, USA, 2448 × 2048 pixels^2^, 10 bits) operating at 50 fps, using its dedicated acquisition software *SpinView*. To enhance contrast, the pool was illuminated in a dark field configuration using a square LED panel (EFFI-SBL, Effilux, France) as a light source, producing a red light (*λ* = 625 nm) to minimize phototaxis [34].

### 4.4. Image processing and analysis

Raw images were directly recorded to a hard drive and compressed by first removing the background then applying a threshold [11]. Automatic tracking was performed on compressed images using *FastTrack* [35]. To maximize tracking quality, tracked movies were then visually inspected and the few remaining errors were manually corrected using the embedded post-processing tools of the *FastTrack* program. At each time *t*, the cell contour was fitted by an ellipse whose orientation *θ*(*t*), defined as the angle between the major axis of the ellipse and the horizontal axis *x* of the images (see figure 1*c*,*d*; electronic supplementary material, section II, figure S2), was computed from the asymmetry of the pixel histogram along the major axis. The semi-major and semi-minor axes were estimated at *a* = 68.5 ± 7.7 μm and *b* = 29.5 ± 4.4 μm, respectively (average ± s.d., *N* = 5080 trajectories).

### 4.5. Post-processing

A Matlab (Mathworks Inc. USA) script was used to remove trajectories shorter than 1 s and time intervals during which the object was immobile. Another Matlab script was used to remove trajectories that presented circling motions, based on a measure of the sign of the instantaneous angular velocity $\dot{\theta }(t)=(\theta (t+\Delta t)-\theta (t))/\Delta t$ with Δ*t* = 20 ms, the time interval between frames.

### 4.6. Detection of avoiding reactions

In our analysis, an AR is described as a two-step process: a BS followed by a reorientation. The BS was detected when the instantaneous motion vector **m**(*t*) and the orientation vector **o**(*t*) (posterior to anterior) pointed in opposite directions, i.e. **m**(*t*) · **o**(*t*) < 0. BS events consisting of a single frame were discarded. The reorientation event was identified as the time interval during which the instantaneous angular speed was large enough, i.e. $|\dot{\theta }(t)|=|\omega (t)|>\omega_{\mathrm{thr}}=150^{\circ }\;s^{-1}$ (see electronic supplementary material, section III and figure S3).

### 4.7. Detection of mechanical contacts

We defined a contact corona of radius *r*_*c*_ = *r*_*p*_ + *δr*, where *δr* = 19 μm was chosen using physical arguments and visual inspections (see electronic supplementary material for details, section VI and figure S5*a*). This sets a contact interaction interval $\left[t_{r_{c}}^{i},t_{r_{c}}^{f}]=\{t\in [t_{i},t_{o}]|r_{\min}(t)<r_{c}\right\}$, where *r*_min_(*t*) is the distance between the centre of the pillar and the ellipse that fits the cell at time *t*, and where *t*_*i*_ (*resp.* *t*_*o*_) is the time at which the cell centre enters (*resp.* leaves) the interaction corona. We considered that the cell collided with the obstacle if and only if its incident angle *θ*_in_ when entering the contact corona was smaller than a threshold, namely $\theta_{\mathrm{in}}(t_{r_{c}}^{i})<{\tilde{\theta }}_{r_{c}}^{i}$, where the contact angle threshold is defined in the same way as for ${\tilde{\theta }}_{i}$ but at $t_{r_{c}}^{i}$ (see electronic supplementary material for details, section VI and figure S5*b*,*c*). In this case, the collision time $t_{c}^{i}$ was defined as the first velocity minimum within the contact interaction interval $\left[t_{r_{c}}^{i},t_{r_{c}}^{f}\right]$ defined above. The interaction was categorized as a SE if no AR was triggered within the contact corona. In this case, the end of a contact was defined as the first time at which the cell is tangent to the obstacle, i.e. $t_{c}^{f}=\min\{t\in [t_{r_{c}}^{i},t_{r_{c}}^{f}]|\theta_{\mathrm{in}}(t)=90^{\circ }\}$. An AR triggered within the contact corona may be an OTAR, or a spontaneous AR occurring shortly after a SE. We used the latency of the AR to determine more finely whether this was an OTAR or a SE (see electronic supplementary material, section VII and figure S6). If it was categorized as an OTAR, the end of the contact was the starting time of the AR. Applying this procedure over all environments allowed us to identify a total of *N*_SE_ = 15 601 SEs, *N*_inst_ = 670 instantaneous OTARs (*τ* ≤ 40 ms) and *N*_delayed_ = 1120 delayed OTARs (*τ* > 40 ms).

### 4.8. Error estimation of the conditional OTAR probability

We denote by *n*_inst_ the number of instantaneous OTARs with collision angle *θ*_*c*_ ± d*θ*_*c*_ and *n*_*c*_ the total number of collisions with *θ*_*c*_ ± d*θ*_*c*_. Then, the number of instantaneous OTARs can be modelled as *n*_*c*_ draws of a binomial distribution of parameter *p* = *n*_inst_/*n*_*c*_ and variance *mp*(1 − *p*). Thus, the conditional probability *p* = *P*(OTAR|*θ*_*c*_) represented in figure 4*b* can be estimated as *n*_inst_/*n*_*c*_ and its error is given by $\sqrt{n_{c}p(1-p)}/n_{c}$. The same estimation was applied for delayed OTARs.

### 4.9. Numerical computations

Data analysis and numerical computations were done with Matlab. The contact duration *T*(*θ*_*c*_) was fitted using the Curve Fitting Toolbox with the nonlinear least-square method. The set of nonlinear equations for *P*_1_ and *P*_2_(*θ*_*c*_), i.e. equations (2.3) and (2.4), was solved using the function *fsolve.m*.

## Data Availability

All data, including images, analysed data and Matlab scripts developed in this work are available from the Dryad Digital Repository: https://doi.org/10.5061/dryad.m63xsj467 [36]. Supplementary material is available online [37].
